# Generation and characterization of *fruitless* P1 promoter mutant in *Drosophila melanogaster*

**DOI:** 10.1080/01677063.2021.1931179

**Published:** 2021-08-02

**Authors:** Megan C. Neville, Alexander Eastwood, Aaron M. Allen, Ammerins de Haan, Tetsuya Nojima, Stephen F. Goodwin

**Affiliations:** University of Oxford, Centre for Neural Circuits & Behaviour, Oxford, UK

**Keywords:** Drosophila, fruitless, mutant, courtship behavior, CRISPR/Cas9-based targeted genome editing

## Abstract

The identification of mutations in the gene *fruitless* (*fru*) paved the way for understanding the genetic basis of male sexual behavior in the vinegar fly *Drosophila melanogaster*. *D. melanogaster* males perform an elaborate courtship display to the female, ultimately leading to copulation. Mutations in *fru* have been shown to disrupt most aspects of the male's behavioral display, rendering males behaviorally sterile. The *fru* genomic locus encodes for multiple transcription factor isoforms from several promoters; only those under the regulation of the most distal P1 promoter are under the control of the sex determination hierarchy and play a role in male-specific behaviors. In this study, we used CRISPR/Cas9-based targeted genome editing of the *fru* gene, to remove the P1 promoter region. We have shown that removal of the P1 promoter leads to a dramatic decrease in male courtship displays towards females and male-specific sterility. We have expanded the analysis of *fru* P1-dependent behaviors, examining male's response to courtship song and general activity levels during12-hour light: dark cycles. Our novel allele expands the mutant repertoire available for future studies of *fru* P1-derived function in *D. melanogaster*. Our *fru^ΔP1^* mutant will be useful for future studies of *fru* P1-derived function, as it can be homozygosed without disrupting additional downstream promoter function and can be utilized in heterozygous combinations with other extant *fru* alleles.

## Introduction

In 1963, Kulbir Gill published a small research note in *Drosophila* *Information Service* entitled *‘A mutation causing abnormal courtship and mating behavior in males of Drosophila melanogaster’*. While looking for male-sterile mutations in *D. melanogaster* by a forward genetic screen, he isolated an X-ray-induced recessive mutation located on the third chromosome (Gill, 1963). The effects of the mutation were male-specific, in that homozygous mutant females showed no noticeable phenotypic or behavioral differences from wild-type (Hall, 1978). Homozygous mutant males, however, exhibited several overt differences from normal male courtship behavior: they did not curl their abdomens at females to attempt copulation and were considered behaviorally sterile, in addition, they courted other mutant males and wild-type males (Hall, 1978). The mutation was given the moniker *fruitless* (*fru*), which seems fitting as mutant males will father no offspring. The identification of this first mutant allele (*fru^1^*) paved the way for understanding the genetic basis of male sexual behavior in the vinegar fly *D. melanogaster*.

Fast forward 20 years, the Wasserman lab, looking for single *P*-element insertion mutants which cause male sterility, again identified mutations which caused males to court other males, and mapped to the *fru* locus (Castrillon *et al*., 1993). These new alleles of *fru* (*fru^3^* and *fru^4^*) made it possible to clone the gene through complementation analysis of mapped deficiencies with these alleles, followed by a chromosomal walk (Ryner *et al*., 1996). A contemporary *P*-element insertion screen, in the Yamamoto lab, focused on identifying behaviorally sterile males, uncovered a different allele of *fru* (*fru^sat^*) which enabled concurrent cloning of the *fru* gene (Ito *et al*., 1996). Collectively these studies unearthed the molecular secrets of the gene, characterizing *fru* DNA and mRNA sequences and determined that it encoded a member of the BTB-Zn-finger family of transcriptional regulators (Siggs & Beutler, 2012). This new collection of *fru* alleles contributed to our initial understanding of how *fru* regulated courtship from the behavioral analysis of males and females carrying mutations at the locus (Villella *et al*., 1997).

Sex in *Drosophila* is governed by the sex determination hierarchy (SDH), where the presence of two X chromosomes sets into motion a sex-specific alternative splicing cascade, leading to male and female sexual differentiation (**reviewed in** Cline & Meyer, 1996). The molecular identification and characterization of *fru* revealed a complex locus with multiple promoters, some of which were essential for viability in both sexes (**reviewed in** Billeter, Rideout, Dornan, & Goodwin, 2006a). The most distal promoter, defined as P1, sits approximately 140 Kb upstream of the general coding region. Transcripts from the P1 promoter, undergo sex-specific alternative splicing and encode the male-specific Fru^M^ proteins (Billeter *et al*., 2006b). These putative transcription factors, containing one of three alternative C_2_H_2_ zinc-finger DNA binding domains, determine many of the neuronal substrates for sexual behaviour in the male central nervous system (CNS) (Neville *et al*., 2014**;** Von Philipsborn *et al*., 2014).

While forward genetic screens remain one of the most powerful tools to study biological pathways in *Drosophila*, the development of reverse genetic approaches, like homologous recombination, permitted the generation or rescue of mutations in genes for which a DNA clone or sequence was available (Rong & Golic, 2000). In 2005, the Dickson lab used homologous recombination to introduce mutations in the *fru* locus which altered the ability of *fru* P1 transcripts to be sex-specifically spliced, forcing Fru^M^ expression in females; these females had been masculinized by Fru^M^ and were able to display many male pre-copulatory courtship behaviors (Demir & Dickson, 2005). Such reverse genetic approaches became more accessible with the advent of CRISPR/Cas-9 technologies, enabling genomic engineering of precise mutations in *D. melanogaster* with relative ease (Bassett, Tibbit, Ponting, & Liu, 2013**;** Gratz *et al*., 2013**;** Yu *et al*., 2013).

In this study, we add to the range of *fru* alleles by generating a deletion of a region spanning the *fru* P1 core promoter via CRISPR/Cas-9 technology, producing a novel allele, *fru^ΔP1^*. We show that Fru^M^ expression is undetectable in our allele. Homozygous mutant males exhibit drastically reduced levels of courtship behavior towards females and are behaviourally sterile. We extended our analysis to examine for the first time the effects of *fru* P1 promoter loss on male activity and their ability to respond to courtship song stimuli. Our *fru^ΔP1^* allele will be useful to the broader scientific community studying the role *fru* plays in defining the male nervous system.

## Materials and methods

### Targeted removal of *fruitless* P1 promoter sequences

CRISPR-Cas9 genome editing was used to remove P1 promoter (first exon) sequences in the *fru* locus generating line *fru^ΔP1^*. Two fragments with homology to the *fru* genomic regions on either side of the P1 promoter were cloned using Gibson Assembly Master Mix (New England Biolabs) with primers for 5′ Fragment: G1_5f and G1_5r and 3′ Fragment: G1_3f and G1_3r (Table S1), into the targeting vector pDsRed-attP (a gift from Melissa Harrison & Kate O'Connor-Giles & Jill Wildonger; Addgene plasmid # 51019) digested with *XhoI* and *NotI*. gRNA expressing constructs pCFD3-Fru5_1 and pCFD3-Fru3_3 were generated in the vector pCFD3-dU6:3gRNA (a gift from Simon Bullock; Addgene plasmid #49410) against target regions Fru5_1 and Fru3_3. Constructs were co-injected into the strain vas-Cas9.RFP- (Bloomington stock #55821), progeny were screened for *DsRed*^+^ expression in the eye. Seven lines were identified, all of which mapped to the 3rd chromosome. PCR and sequencing were used to confirm the deletion of P1 sequences. *DsRed* was removed from two independently isolated lines (*fru^ΔP1.1^* and *fru^ΔP1.2^*) by crossing to a Cre recombinase constitutively expressing line (Bloomington stock #851).

### RT-PCR analysis

RNA was extracted from four biological replicates of *Canton S* and *fru^ΔP1^* whole adult flies (*n* = 10 flies per extract) using TRIzol™ (ThermoFisher, cat# 15596026) following the manufacturer’s directions. cDNA was generated with the SuperScript™ III First-Strand Synthesis System (ThermoFisher, cat# 18080051) following the manufacturer’s directions, with 1 µg input RNA and primed with oligo(dT). Primer sequences were designed with Primer3 in Geneious 8.1.7 software (Kearse *et al*., 2012). Potential for off-target priming was evaluated with Primer-Blast (Ye *et al*., 2012). Amplicon secondary structure was evaluated with MFold web tool (Zuker, 2003). Quantitative PCR was carried out using the Roche™ LightCycler® 480 with the LightCycler® 480 SYBR Green I Master kit (cat# 04707516001). Cycling conditions were performed following manufacturer’s recommendations with 60 °C annealing temperature for 15 s, and a 15 s extension time. Data analysis was performed with qbase + software, version 3.2 (Biogazelle, Zwijnaarde, Belgium - www.qbaseplus.com). Primer efficiencies were calculated from amplification of serial dilutions of a pooled cDNA template. Four technical replicates were run for each biological replicate across each primer set. Technical replicates with a difference in Cq value of greater than 0.5 were discarded. A panel of potential reference genes was selected based on previous studies (Ling & Salvaterra, 2011; Ponton, Chapuis, Pernice, Sword, & Simpson, 2011). Reference gene stability was evaluated with geNorm (Vandesompele, De Paepe, & Speleman, 2002). Reference genes with an M-value of greater than 0.2 or a coefficient of variation (CV) of greater than 0.2 were discarded. This yielded 4 reference genes (*14–3-3ε*, *RpL32*, *Su(Tpl)*, and *eIF1A*) with a combined M-value of 0.181 and a CV of 0.083. Expression of *fru^M^* was then normalized using multi-gene normalization (Hellemans, Mortier, De Paepe, Speleman, & Vandesompele, 2007). Sample were collected and processed following abbreviated MIQE recommendations (Taylor, Wakem, Dijkman, Alsarraj, & Nguyen, 2010).

### Courtship behavior

Flies were raised at 25 °C in a 12 h:12 h light: dark cycle. Individual virgin males were collected and aged for 5–7 days post-eclosion while virgin females were aged for 3–5 days post-eclosion at 25 °C and assays were carried out at 25 °C within 1–4 h of the commencement of the light cycle. To score courtship, individual naïve males were introduced into a round chamber (19 mm diameter × 4 mm height) with an individual wild-type *Canton S* female. The following parameters were measured during a 60 min observation period: courtship initiation (first bout lasting over 3 s that included two or more behavioral displays including following, tapping, wing extension, licking, and attempted copulation), time to copulation (in minutes), copulation success (% copulating in 1 h), and courtship index.

% Fertility is the proportion of females that produce viable progeny. Males and females tested for fertility were collected at eclosion, stored in groups of 3–5 and aged for 5 days. They were then introduced individually into food vials containing three wild-type virgin females or males aged 3–5 days. All vials were scored for presence of larval progeny after 10 days. Vials containing a dead experimental male or female were discounted.

### Male song analysis

For recording song, experimental male flies were paired with a wild-type female in cylindrical courtship chambers with a diameter of 10 mm and a height of 4 mm. Sound was recorded with a CMP5247TF-K microphone in an Insectavox (Gorczyca & Hall, 1987). Recordings were analyzed using the MATLAB toolbox FlySongSegmenter (Arthur, Sunayama-Morita, Coen, Murthy, & Stern, 2013). Sine song was excluded from our analysis sine song detection in *fru^ΔP1^* mutant males using FlySongSegmenter was inconclusive. Each assay was performed within 3 h of the commencement of the light cycle.

### Song playback male chaining assay

Our chaining protocol was carried out as described in Inagaki, Kamikouchi, and Ito (2010**)**. Virgin males were collected within six hours post-eclosion and muted via cutting of both wings rostral to the anterior crossvein. Subjects were then housed in groups of six to eight animals of the same genotype in Perspex vials containing food for 3–7 days. For each assay, six age-matched subjects of the same genotype were transferred into a single rectangular-shaped behavioural chamber (Plexiglass: 50 mm x 10 mm x 6 mm). Four chambers were loaded with different genotypes and recorded simultaneously. Each assay was performed within 3 h of the commencement of the light cycle in an acoustically-attenuated experimental room at 25 °C, between 40–50% relative humidity. Playback of auditory stimuli was delivered from a loudspeaker (Mach sub bass speaker) positioned 10 cm away from the chamber set. As we use naturalistic playback stimuli the amplitude within playback varies between 85 and 100 dB, which was controlled using a CEL-246 sound-level meter. We used an EP-800 amplifier, which was connected to a creative sound blaster X-Fi Xtreme audio PCI sound card in an OptiPlex 3020 mini tower PC. Windows Media Player (version 12.0.7601.19148; default settings) was used to control playback. The chamber set was backlit from underneath using an LED light box (ComicMaster Tracer LED-A4, Too Marker Products, Japan) in order to maximise contrast for visualisation of behavioural interactions. Flies were observed for 5-min in the absence of any sound, and then for a subsequent 5-min in the presence of an acoustic stimulus. Behaviour was recorded by video using a monochrome digital camera (Stingray F-033C camera) NI PCIe-8253, IEEE 1394 b Board with Vision Acquisition using its zoom lens (Lametar 2.8/25 mm, Jenoptik GmbH, Jena, Germany). Each video file was sampled at 1 frame per second. Chaining indices were generated by analysis with ‘ChaIN’ automated tracking software as previously described by Yoon *et al*. (2013**).**

### Male locomotor analysis

Virgin male flies were collected within six hours post-eclosion and housed in vials of six to eight flies of the same genotype for 3 days prior to being loaded into glass tubes (6 mm x 0.5 mm) which contained 1 cm of agar food. Flies were placed in incubators at 25 °C in a 12-h light: dark cycle. Flies were first allowed to habituate to the single-housing condition and apparatus for two days. On day 3–5 locomotor activity was assessed using *Drosophila* Activity Monitors (Trikinetics) that recorded interception of an infrared beam halfway along the tube. This registration of a beam break was used as a proxy for activity. Subjects that did not register beam breaks throughout this period were deemed to have died and were excluded from analysis.

### Statistics

All statistical analyses were carried out using Prims 8.4.3.

### Immunohistochemistry and image analysis

Adult brain dissection and staining were carried out as described previously (Lee *et al*., 2000). Using a primary antibody solution of rabbit anti-Fru^M^ (1:500) for 24 h at 4 °C. After washes, tissues were stained in a secondary antibody solution containing 1:500 of an anti-Rabbit secondary Alexa 488, (1:500 Invitrogen).

## Results

### Generation of P1 promoter deletion in the *fruitless* locus

Our intension was to disrupt the core P1 promoter region in *fruitless* since the SDH regulates only transcripts from this promoter. We first examined the transcriptional start site (TSS) of the P1 promoter between closely related *Drosophila* species (Figure S1). We found the TSS sequence annotated in Flybase matches the Initiator (Inr) motif consensus associated with ‘sharp’ initiation (Bhardwaj, Semplicio, Erdogdu, Manke, & Akhtar, 2019), common in adult tissue-specific genes and terminally differentiated cell-specific genes (Haberle & Stark, 2018). Examination of male and female head Cap Analysis Gene Expression (CAGE) data, available through ModEncode (modENCODE CAGE), reinforced sharp initiation near this TSS in both sexes. Species comparisons identified a downstream promoter element (DPE; Figure S1) and a lack of an exact TATA-binding sequence upstream of the TSS. The *fru* P1 core promoter appears to be what is classified as a type-2 promoter by modENCODE, containing an Inr and DPE, with no recognisable TATA-box region (Chen *et al*., 2014).

**Figure 1.**
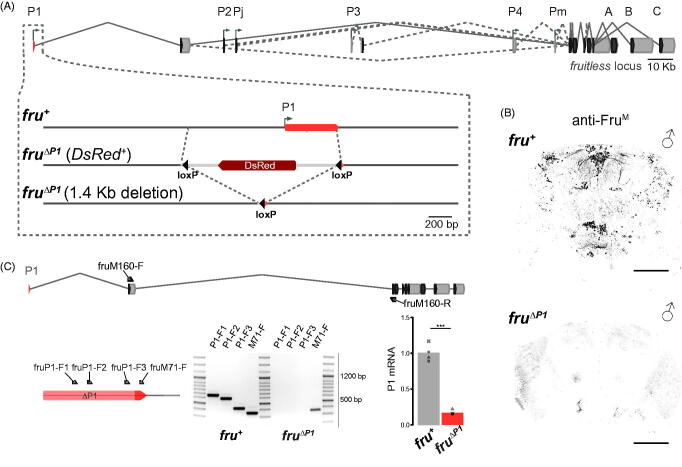
Generation of novel *fru^ΔP1^* mutant. (A) Schematic depicting the generation of *fruitless* P1 core promoter deletion. The P1 promoter was replaced by *DsRed* sequences flanked by *loxP* recombination sites using CRISPR-Cas9 genome editing, resulting in the P1 deletion line *fru^ΔP1^*. (B) Immunohistochemistry analysis of Fru^M^ protein expression in *fru^ΔP1^*homozygous males and wild-type control males. Adult Brain (anterior view, dorsal up) Scale bars represent 50 µm. (C) RT-PCR of *fru^ΔP1^*. Schematic of the *fru* locus (above), exons are grey with CDS in black, the P1 exon shown in orange and primers (fruM160-F and fruM160-R) used for qPCR in black. Magnified region of exon 1 (below, left), with primer binding sites annotated above the exon, and the deletion within the exon annotated. End-point RT-PCR of CS and *fru^ΔP1^* using the four primers shown on the left, with reverse primer fruM160-R shown above. The forward primers are marked above the gel image and the genotypes are marked below the gel image. Note the fruM7-1F primer binds to the region of *fru* P1 exon remaining in *fru^ΔP1^*. The genotypes are separated by 100 bp ladders with size markings on the right. (Bottom, right) RT-qPCR of *fru* P1 mRNA (using primers fruM160-F and fruM160-R), and normalized using delta-delta-Ct to 4 separate reference genes. The bars represent the mean of the four biological replicates which are overlaid (***p < 0.001).

To generate a *fru* P1 promoter deletion we adopted the CRISPR-homology directed gene targeting approach (Gratz *et al*., 2014). We targeted a 1.4 Kb region of the *fru* locus for removal, which extended from approximately 1 Kb upstream of the TSS, removing sequences beyond the core promoter, to 400 bp downstream of the TSS, just before the end of the first P1 exon (Figure 1(A)). Initially, we used the red fluorescent eye marker *DsRed* to screen for successful recombination events, generating *fru^ΔP1^* (*Ds+*) alleles. Using Cre recombinase, we removed the Lox-flanked DsRed marker to generate *fru^ΔP1^* alleles (Siegal & Hartl, 1996). We backcrossed these alleles for eight generations into a wild-type *Canton S* genetic background before further analysis.

We initially molecularly characterized putative *fru^ΔP1^* alleles to verify recovery of the intended genome modification (see methods). Bona fide *fru^ΔP1^* alleles were tested with immunohistochemistry analyses to examine the expression of the male-specific Fru^M^ protein generated from mRNA transcripts derived from the P1 promoter in the brain (Lee & Hall, 2001). We presumed that *fru^ΔP1^* homozygous adult males would lack detectable Fru^M^, and indeed males homozygous for the *fru^ΔP1^* allele, in comparison to *Canton S* (+/+) controls, were null for Fru^M^ immunostaining (Figure 1(B)). We next examined the expression of RNA transcripts associated with the P1 promoter region in *fru^ΔP1^* homozygous adult males and control *Canton S* males. *fru^ΔP1^* males did not show any amplification of *fru* products from the deleted region, while control *Canton S* (*CS*) males showed amplification of expected RNA products. As a small portion of the first P1 exon was not deleted in the *fru^ΔP1^* allele, we surprisingly found amplification of RNA transcripts containing this portion of the exon that successfully spliced into the next downstream *fru* exon (M71-F in Figure 1(C)). We next quantified the transcription levels of these detected RNA transcripts using RT-qPCR, and found an 83% reduction of RNA transcript levels in *fru^ΔP1^* homozygous whole fly RNA extracts (P1 mRNA in Figure 1(C)). These data show that although an extended region including the TSS and core promoter region of P1 had been deleted, transcription was not abolished, however as whole flies were used for these analyses the cell/tissue specificity of this transcription is unclear. Our lack of anti-Fru^M^ antibody expression in the brain suggests that protein levels in this tissue are either below the level of detection or are found in other tissues outside the brain.

### Courtship behavioural defects of *fru^ΔP1^* mutant males

**Figure 2.**
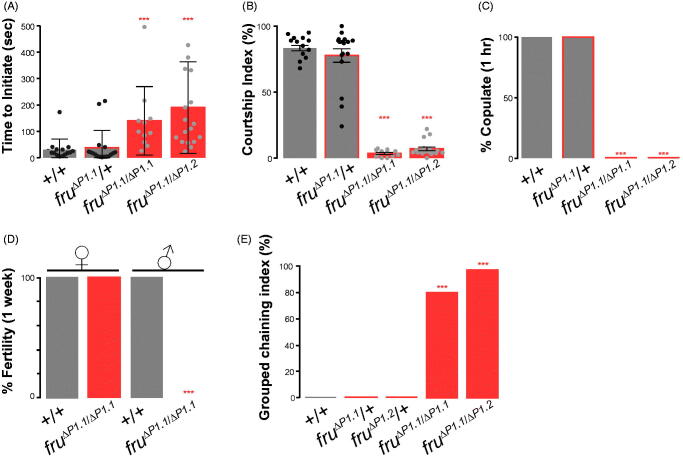
Courtship behaviour of *fru^ΔP1^* mutants. (A) Time to initiate. ****p < 0.0001 (Kruskal-Wallis and post-hoc Dunn’s test). (B) Courtship indices. ****p < 0.0001 (Kruskal-Wallis and post-hoc Dunn’s test). (C) Percentage of males mating within 1 hr. ***p < 0.0001 (Fisher’s exact test). (D) Male and female fertility. Percent fertile *fru^ΔP1^*homozygous mutant and control males and females. ***p < 0.0001 (Fisher’s exact test). (E) Grouped chaining indices is a measure of the percent of time three of more males formed chains over a 10-minute observation period. ***p < 0.0001 (Fisher’s exact test). (A–C) All genotypes indicated are males; target females are *Canton S*. *N* = 16–18. Error bars indicate SEM. *N* = 20 in (D).

To examine the effect of losing core promoter-driven expression of *fru* P1 derived mRNA transcripts, the interactions of males carrying the *fru^ΔP1^* mutation with control females were studied. When placed with a female, *fru^ΔP1^* mutant males displayed severe behavioral deficiencies: mutant males take over six times as long to initiate courtship (Figure 2(A)), and once initiated, the levels of courtship displayed (Courtship Index) were approximately 80 times lower than control males (Figure 2(B)). During the 1-h observation period, *fru^ΔP1^* homozygous mutant males never copulated (Figure 2(C)), rendering them behaviorally sterile, the defining phenotype of initial studies of mutants at the *fru* locus (Hall, 1978). Over a week, mutant male sterility was indisputable, whereas females homozygous for the *fru^ΔP1^* allele were fertile (Figure 2(D)). We note that *fru^ΔP1^*/+ heterozygous males behaved as *Canton S* (+/+) controls; therefore, no dominant phenotypes were observed (Figure 1(A–C)). We confirmed that males heterozygous for two independently isolated *fru^ΔP1^* mutant alleles (*fru^ΔP1.1^*/*fru^ΔP1.2^*) displayed the same mutant phenotype as the homozygous allele (*fru^ΔP1.1^*/*fru^ΔP1.1^*) (Figure 1(A–C)).

Previous studies have observed robust *fru* mutant male-male courtship which emerges by grouping mutant males over several days, where males court other males, while also being courted themselves, thereby forming courtship chains (Hall, 1978**;** Villella *et al*., 1997). We grouped 3-day old control, heterozygous or homozygous *fru^ΔP1^* mutant males on food and examined chaining behavior after four days (Figure 2(E)). We quantified a grouped chaining index, based on the percent of time three of more males formed chains over a 10-min observation period. We found that males homozygous for *fru^ΔP1^* mutant alleles showed robust levels of male-male chaining, with at least one chain of 3 or more males observed over 80% of the 10 min (Figure 2(E)). We demonstrated that the removal of the *fru* P1 promoter region is sufficient to induce robust levels of male-male courtship when group-housed, while single male-female pairings showed significantly reduced levels of male courtship behaviour.

### *Fru^ΔP1^* mutant males produce low levels of defective song

**Figure 3.**
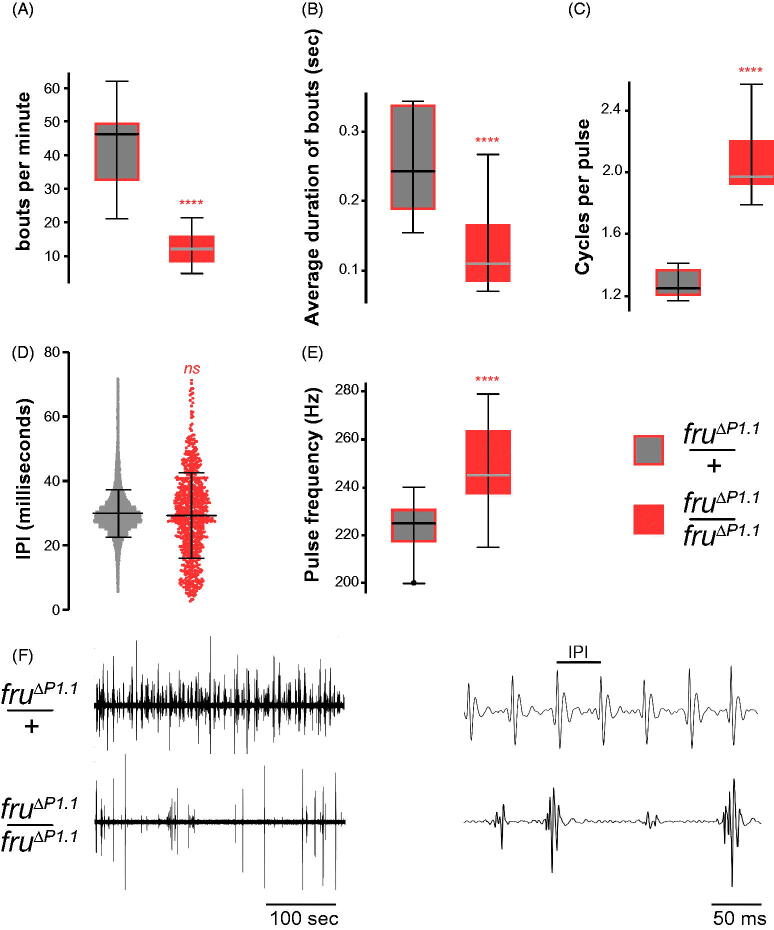
*fru^ΔP1^* mutant analysis of male courtship song. (A) Pulse Bouts Per Minute. (Mann-Whitney test). (B) Average Duration of bouts (sec). (Mann-Whitney test). (C) Number of cycles per pulse. (Nested *t*-test based on genotype). (D) Interpulse interval in ms. (Nested *t*-test based on genotype). (E) Pulse frequency in Hz. (Nested *t*-test based on genotype). (F) Examples of pulse song trains in *fru^ΔP1^* mutants. (A–C) and (E) Boxplots of individual flies, median with interquartile range indicated by box, whiskers represent 5 and 95 percentiles. (D) Scatter dot plot with median and SEM. (A–E) ****p < 0.0001, ns = not significant. All genotypes indicated are males; target females are wild-type *Canton S*. N = 9 flies recorded.

We examined the ability of *fru^ΔP1^* mutant males to produce courtship song when paired with a female. Song recordings were analysed using FlySongSegmenter (Arthur *et al*., 2013). We found that the *fru^ΔP1^*/+ heterozygous control male song appeared normal, while *fru^ΔP1^* mutant males produced low levels of pulse song with approximately a quarter the number of pulse bouts per minute, with the duration of pulse bouts significantly shorter (Figure 3(A,B)). *fru^ΔP1^* mutant males generated a significantly higher number of wave cycles per pulse (Figure 3(C)); the unusual form of the pulse shape is apparent when looking at the traces of the pulses themselves (Figure 3(F)). Interestingly, one of the two key species-specific parameters associated with the song, time between pulses or interpulse intervals (IPI) was not significantly different from the *fru^ΔP1^*/+ heterozygous control (Figure 3(D)); however, the broad range of IPIs observed shows this parameter is highly variable in the *fru^ΔP1^* mutant males. In contrast, the other species-specific parameter, pulse frequency, was significantly longer and highly variable (Figure 3(E)). We did not examine Sine song as although it was clearly detected in control male song, we could not reliably distinguish it from background signals in *fru^ΔP1^* mutant male song using FlySongSegmenter. Overall our data confirm the long-established role of *fru* P1 derived transcripts in the production of robust male courtship song.

### *Fru^ΔP1^* mutant response to auditory stimuli song perception

Early studies investigating the role of male courtship song found that playing simulated courtship song to groups of males evoked male-male courtship behaviours (Von Schilcher, 1976). When courtship song was played to groups of muted males (amputated wings), they increased their locomotor activity and started courting each other, forming courtship chains. The role of *fru* in the perception of this courtship-relevant stimuli had not been investigated before.

**Figure 4.**
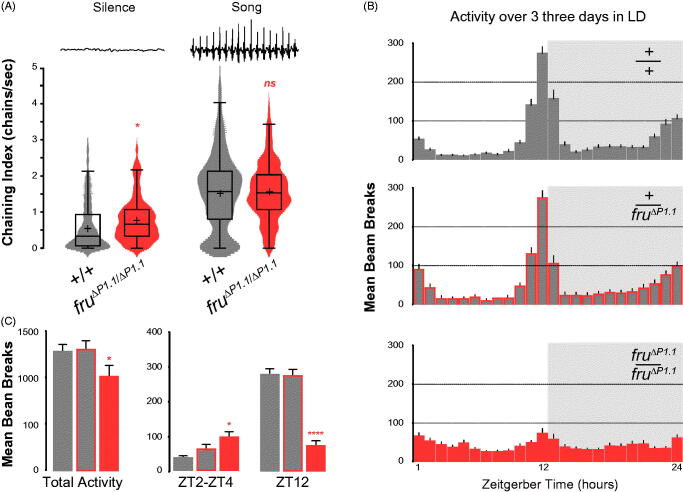
*fru^ΔP1^* males’ general activity and response to courtship song. (A) Distributions of chaining behaviour exhibited by males during 5-minute silence and 5-minute playback of male courtship song. Violin plots show distribution of data about the mean (plus sign) and median (centre line of box and whisker plot overlay). *p < 0.01 Wilcoxon signed-rank test; *p* < 0.0001 within genotypes when comparing song and song playback conditions. *N*(+/+)=29, *N*(*fru^ΔP1.1^*/*fru^ΔP1.1^*)=21. (B) Male locomotor activity over 3 consecutive days in 12-hr light: dark (LD) cycle measured as the number of beam crosses in 1-hr intervals. A 2-way ANOVA found no significance based on genotype (*p* = 0.0929). (C) Male locomotor activity over 3 consecutive days in 12-hr light: dark cycle in total, during behavioural testing period (ZT2-ZT4) and the hour prior to lights-off (ZT12). A ordinary one-way ANOVA was performed, followed by Tukey’s multiple comparisons test. *p < 0.01, ****p < 0.0001, ns = not significant. (B, C) Error bars indicate SEM. *N*(+/+)=40, *N*(+/*fru^ΔP1.1^*)=38, *N*(*fru^ΔP1.1^*/*fru^ΔP1.1^*)=28.

To investigate the response of *fru^ΔP1^* mutant males to courtship song playback, muted males were introduced into a chamber where they were played courtship song (see materials and methods). Analysis of male-male chaining was automated using the software ‘ChaIN’ (Yoon *et al*., 2013), which generates a chain index based on the number of animals forming individual male-male chains per video frame (1 frame/second). During the initial 5-min of silence, both grouped *fru^ΔP1^* homozygous mutant males and *Canton S* (+/+) males exhibited low-levels of chaining, however, *fru^ΔP1^* mutant males displayed small but significantly higher levels (*p* < 0.01) (Figure 4(A), left). We next examined the response of *fru^ΔP1^* homozygous mutant males and wild-type males to song playback. We found song playback evoked robust levels of chaining, with no significant differences in the levels between the genotypes (*p* = 0.61948) (Figure 4(A), right). These findings suggest that *fru^ΔP1^* mutant males can perceive courtship song and process this sensory information to produce the same motor output response as wild-type males in this assay.

### Locomotor activity of *fru^ΔP1^* mutant males

To establish if any of the *fru^ΔP1^* mutant male phenotypes are due to general defects in their locomotor activity, we set out to establish the general activity of these males over 3-days in a 12:12 h light: dark cycle. Individual *fru^ΔP1^* mutant males, *fru^ΔP1^*/+ heterozygous and *Canton S* (+/+) control males were observed (Figure 4(B)). We found that *fru^ΔP1^* mutant males over this observation period showed lower levels of activity, especially apparent was the differences in the magnitude of the peak of activity before lights-off (Figure 4(C) left and right, respectively). We specifically examined the time of day during which our behavioural assays were carried out (ZT2-ZT4) to determine if any of our observed phenotypes were due to changes in locomotor activity. We found locomotion of *fru^ΔP1^* mutant males was higher during this period compared to *Canton S* and *fru^ΔP1^* heterozygous mutant males (Figure 4(C) middle); therefore, lack of courtship displays during this period are not associated with a lack of locomotor activity; however, the increase in chaining observed during song playback assays may have been influenced by this difference in activity levels. Our results suggest that *fru* P1 derived products play a role in the gating of baseline locomotor activity, showing less variation in the peaks and troughs of activity over a 24-h period in light: dark conditions when compared to controls.

## Discussion

Our *fru* P1 promoter deletion is the first allele specifically targeting transcription initiation of sex-specifically spliced transcripts from the *fru* locus. Historically various *fru* mutant alleles and heterozygous deficiency combinations were utilized to disrupt *fru* P1 function specifically, without also affecting other *fru* promoters, some of which are essential for viability (Anand *et al*., 2001**;** Ito *et al*., 1996**;** Ryner *et al*., 1996**;** Villella *et al*., 1997). Genetically engineered alleles have primarily focused on sex-specific splicing of the second exon of P1 derived transcripts, targeting the male-specific translation initiation codon (Manoli *et al*., 2005**;** Mellert, Knapp, Manoli, Meissner, & Baker, 2010; Stockinger, Kvitsiani, Rotkopf, Tirián, & Dickson, 2005). We and others, however, have found that targeting this region can cause exon-skipping, which leads to expression of P1 derived protein in both males and females that lack the male-specific N-terminus encoded in the skipped exon (M. Neville unpublished observation; Manoli *et al*., 2005**;** Ferri, Bohm, Lincicome, Hall, & Villella, 2008). Our novel *fru^ΔP1^* mutant, targeted to the promoter region, will be useful for future studies of *fru* P1-derived function, as it can be homozygosed without disrupting downstream promoters, and can be used in heterozygous combinations with other extant *fru* alleles.

A core promoter is a complex DNA element that facilitates the recruitment of the basal transcriptional machinery, including RNA polymerase II (PolII), to the TSS (Vo Ngoc, Kassavetis, & Kadonaga, 2019). Regulatory factors which bind enhancers can distinguish between different promoters, at least in part, through interactions with general transcription factors recruited by core promoter elements, such as the DPE found downstream of the *fru* P1 TSS (Vo Ngoc *et al*., 2019). Enhancer-promoter specific DPE interactions are crucial to ensuring precise spatial and temporal gene regulation of key developmental genes, including the homeotic (Hox) genes in *Drosophila* (Juven-Gershon, Hsu, & Kadonaga, 2008). The traditional view of enhancer-core promoter control on gene expression has been challenged, as many transcripts initiate outside of the classic promoter region, including from within enhancers themselves (Haberle & Stark, 2018). The initiation of transcripts in the absence of 1.4 Kb *fru* P1 promoter region shows that transcription can initiate in the absence of these sequences, perhaps through initiation events at upstream enhancers (Rennie *et al*., 2018). Our finding makes it difficult to precisely determine which sequences need to be deleted from the *fru* locus to abolish all P1 derived transcripts. Extending our current deletion to include the entire first exon would unlikely decrease transcription initiation; any transcripts made would be missing the splice donor site and thus presumably be unable to splice into downstream exons, how this would affect the further processing of these transcripts would need to be determined. Future investigations will be necessary to determine how all *fru* P1-driven expression can be fully removed; these findings should be taken into consideration when engineering mutations designed to disrupt *fru* P1-driven transcription in related *Drosophila* species.

The most overt and essential phenotype associated with *fru* sex-specific expression is the inability to copulate, as it is this deficit that makes them behaviourally sterile and is the characteristic phenotype that led to its initial discovery (Hall, 1978). All *fru* alleles which significantly disrupt *fru* P1 function, including ours, are male sterile. A dichotomy has always existed when defining the role *fru* plays in male pre-copulatory courtship behaviors, in that many mutations which disrupt *fru* P1-derived expression result in a severe decrease in male courtship behavior towards females, while at the same time cause an increase in male courtship towards other males. The proposed role of *fru* as the master regulator of male courtship is less about the ability of males to court and more about the regulation of the stimulation to court along with the fine-tuning the motor outputs of the male's display, such as song production. The expression of Fru^M^ in the nervous system confirms this role, as it is expressed in sensory and central processing neurons, some of which change their neurite morphologies and/or cell numbers depending on its expression (reviewed by Billeter *et al*., 2006a). Fru^M^ expression does not act as a switch, which turns a female into a male nervous system; instead, it acts on a mostly unisex nervous system to enhance and fine-tune male-specific behavioral needs. Indeed, we have previously shown that the activation of only a small number of neurons in the female brain enables them to perform much of the male pre-copulatory courtship display (Rezával *et al*., 2016).

We have shown for the first time in this study *fru* P1 mutant male responses to courtship song stimuli. It has been previously shown that the *fru*-expressing posterior neuronal cluster P1, a male-specific subset of the male pC1 cluster, can respond to male courtship song, as does the female pC1 cluster (Zhou *et al*., 2015). The *fru*-P1neuronal cluster itself gates the male's response to song along with various courtship-relevant stimuli (Clowney, Iguchi, Bussell, Scheer, & Ruta, 2015**;** Kallman, Kim, & Scott, 2015; Kohatsu, Koganezawa, & Yamamoto, 2011). We have shown that in the absence of most *fru* P1-transcripts, males still show a robust response to song stimuli which translates into high levels of male-male courtship; therefore, the feminized *fru^ΔP1^* nervous system is not only capable of song perception but also transducing this signal into motor output. This finding confirms that males are poised to display courtship behaviors in the absence of Fru^M^, and can indeed do so with sufficient levels of stimulation.

In this study, we uncovered a novel male *fru* mutant phenotype when examining activity levels over a 12-h light: dark cycle. A previous study linked male mating drive to activity and sleep, both of which appear to decrease in the absence of *fru* P1 expression (Chen *et al*., 2017). Our study confirms a small cumulative decrease in activity over 24 h, however, our detailed analysis of activity over this period uncovered a dramatic decrease in rhythmic changes in activity in response to the light: dark cycle compared to controls, which show robust peaks and troughs of activity. Our novel allele will aid in follow-up experiments aimed at uncovering the basis of this phenotype, as it can be exploited to directly target *fru* P1-transcripts without necessitating complex allelic combinations which complicate the analysis of activity levels. We note that *fru* is known to be highly expressed in the visual system (Davis *et al*., 2020). Follow-up experiments should focus on examining any visual contributions to this phenotype as well as examine if sleep deprivation might also play a role. It will be interesting to directly link the observed changes in activity to male sexual behavior, including identifying the underlying neuronal circuity and the role *fru* plays in defining its function.

## Supplementary Material

Supplementary_Materials.pdf
