# Supplementary material for: Generation and characterization of fruitless P1 promoter mutant in Drosophila melanogaster
Source: J Neurogenet. 2021 Aug 2;35(3):285–94. doi: 10.1080/01677063.2021.1931179 (PMC8477730; doi:10.1080/01677063.2021.1931179)
Supplement: Supplementary_Materials.pdf [file INEG_A_1931179_SM7418.pdf]

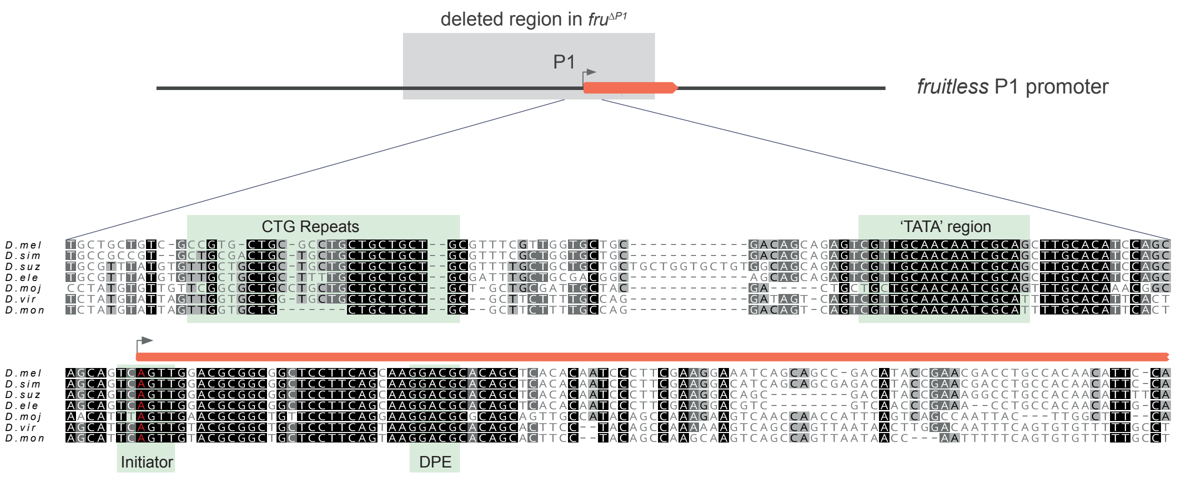

Figure S1. Conserved features of *fru*^{P1} core promoter region.

(Top) A close up of *fru*^{P1} genomic region, highlighting the region deleted in *fru*$^{\Delta P1}$. (Bottom) Alignment of sequence surrounding the *fru*^{P1} TSS in *D. melanogaster* (*D.mel*), *D. simulans* (*D.sim*), *D. suzuki* (*D.suz*), *D. elegans* (*D.ele*), *D. virilis* (*D vir*), *D. montana* (*D.mon*). Shown are 100 nt upstream and downstream of the *D. mel* TSS (in gray). CTG repeats, 'TATA' region, Inr and DPE regions highlighted.

Table S1. Primer and gRNA target region sequences

Primer Name	Sequence
G1_5f	GCATTTAGAATAAATTTTGTGTCGCCCTTGAACTCGATTGACGGAAGAGCCTGATTACGCTTCATTGCTGGC
G1_5r	GAGCACTAGTAAAGATCTCCATGCATAAGGCGCGCCTAGGCCTTCTGCAGCTGCCCCTACGGGAATGACAACC
G1_3f	GCACTACGATCGCAGGTGTGCATATGTCCGCGGCCTCTGCTGAGTGCAT
G1_3r	GCTGAAGCAGGTGGAATTCTTGCATGCTAGCAGATGTAAGAGTACTCAAATATATTATTGAATATAAATT
1433e-F	CCAGCGATATTGCCATGAA
1433e-R	TCCGGCGAGTTGAGAATC
eIF1A-R	GCCCTGGTTAATCCACACC
eIF1A-F	GTCGTCTGGAGGCAATGTG
RpL32-F	CGGATCGATATGCTAAGCTGT
RpL32-R	CGACGCACTCTGTTGTCG
Su(Tpl)-R	GCACTCGTTGTGGAAGTAGACA
Su(Tpl)-F	AAAACAAACTCACATACAACCAAAAA
fruM160-F	AACATCGAAACGGATGTGC
fruM160-R	CGCTCCTTGGTCAGTGTTG
fruM71-F	CAAGTTGCAGCCGCATAAG
fruM71-R	GTGACGTCGCCATCATTTT
fruP1-F1	CGCTTACATAAATGCTAGAGCGC
**gRNA Target Region**	**Sequence**
Fru5_1	TCATTCCCGTAGGGGCAATGAGG
Fru3_3	TGCACTCAGCAGAGGCTGACTGG
